# *Arabidopsis* ENHANCED DISEASE SUSCEPTIBILITY1 promotes systemic acquired resistance via azelaic acid and its precursor 9-oxo nonanoic acid

**DOI:** 10.1093/jxb/eru331

**Published:** 2014-08-11

**Authors:** Finni Wittek, Thomas Hoffmann, Basem Kanawati, Marlies Bichlmeier, Claudia Knappe, Marion Wenig, Philippe Schmitt-Kopplin, Jane E. Parker, Wilfried Schwab, A. Corina Vlot

**Affiliations:** ^1^Helmholtz Zentrum Muenchen, Department of Environmental Sciences, Institute of Biochemical Plant Pathology, Ingolstaedter Landstr. 1, D-85764 Neuherberg, Germany; ^2^Technical University Munich, Biotechnology of Natural Products, Liesel-Beckmann-Str. 1, D-85354 Freising, Germany; ^3^Helmholtz Zentrum Muenchen, Department of Environmental Sciences, Research Unit Analytical Biogeochemistry, Ingolstaedter Landstr. 1, D-85764 Neuherberg, Germany; ^4^Max-Planck Institute for Plant Breeding Research, Department of Plant-Microbe Interactions, Carl-von-Linné-Weg 10, D-50829 Cologne, Germany

**Keywords:** *Arabidopsis thaliana*, azelaic acid, EDS1, lipid peroxidation, 9-oxo nonanoic acid, systemic acquired resistance.

## Abstract

Systemic acquired resistance (SAR) is a form of inducible disease resistance that depends on salicylic acid and its upstream regulator *ENHANCED DISEASE SUSCEPTIBILITY1* (*EDS1*). Although local *Arabidopsis thaliana* defence responses activated by the *Pseudomonas syringae* effector protein AvrRpm1 are intact in *eds1* mutant plants, SAR signal generation is abolished. Here, the SAR-specific phenotype of the *eds1* mutant is utilized to identify metabolites that contribute to SAR. To this end, SAR bioassay-assisted fractionation of extracts from the wild type compared with *eds1* mutant plants that conditionally express *AvrRpm1* was performed. Using high-performance liquid chromatography followed by mass spectrometry, systemic immunity was associated with the accumulation of 60 metabolites, including the putative SAR signal azelaic acid (AzA) and its precursors 9-hydroperoxy octadecadienoic acid (9-HPOD) and 9-oxo nonanoic acid (ONA). Exogenous ONA induced SAR in systemic untreated leaves when applied at a 4-fold lower concentration than AzA. The data suggest that *in planta* oxidation of ONA to AzA might be partially responsible for this response and provide further evidence that AzA mobilizes *Arabidopsis* immunity in a concentration-dependent manner. The AzA fragmentation product pimelic acid did not induce SAR. The results link the C9 lipid peroxidation products ONA and AzA with systemic rather than local resistance and suggest that EDS1 directly or indirectly promotes the accumulation of ONA, AzA, or one or more of their common precursors possibly by activating one or more pathways that either result in the release of these compounds from galactolipids or promote lipid peroxidation.

## Summary

Compromised systemic acquired resistance in *eds1* mutant *Arabidopsis* plants is associated with a reduced ability of the mutant to accumulate azelaic acid and its precursor 9-oxo nonanoic acid.

## Introduction

Plants protect themselves from pathogen invasion by innate immune mechanisms. In dicotyledonous plants, for example *Arabidopsis thaliana*, defence against biotrophic pathogens is dependent on the phytohormone salicylic acid (SA) and can be divided into local and systemic phases of immunity (Vlot *et al.*, 2009; Spoel and Dong, 2012; Fu and Dong, 2013). Locally, plants respond to pathogen-associated molecular patterns (PAMPs) with PAMP-triggered immunity (PTI; Jones and Dangl, 2006). Alternatively, the recognition of pathogen effectors leads to effector-triggered immunity (ETI), which augments PTI (Tsuda *et al.*, 2009; Tsuda and Katagiri, 2010). In contrast to PTI, ETI often results in hypersensitive response (HR)-associated death of the infected site and surrounding cells (Jones and Dangl, 2006; Maekawa *et al.*, 2011). In ETI, pathogen effectors are recognized by plant nucleotide-binding leucine-rich repeat (NLR) receptors the majority of which possess N-terminal Toll-Interleukin1 Receptor-like (TIR) or coiled-coil (CC) domains, referred to as TNLs and CNLs, respectively (Maekawa *et al.*, 2011; Bonardi and Dangl, 2012). PTI and ETI are associated with SA accumulation and a burst of reactive oxygen species (ROS; Jones and Dangl, 2006), and induce SA-dependent systemic acquired resistance (SAR) in systemic uninfected tissues (Cameron *et al.*, 1994; Mishina and Zeier, 2007; Vlot *et al.*, 2009; Liu *et al.*, 2010; Fu and Dong, 2013; Breitenbach *et al.*, 2014).

Long-distance acting metabolites reported to be associated with SAR include methyl salicylate (Park *et al.*, 2007), the diterpenoid dihydroabietinal (Chaturvedi *et al.*, 2012), the non-protein amino acid pipecolic acid (Navarova *et al.*, 2012), the C9 dicarboxylic acid azelaic acid (AzA; Jung *et al.*, 2009), and glycerol-3-phosphate (G3P; Chanda *et al.*, 2011). In addition, the lipid transfer proteins AZELAIC ACID INDUCED 1 (AZI1; Jung *et al.*, 2009; Yu *et al.*, 2013), DEFECTIVE IN INDUCED RESISTANCE1 (DIR1), and DIR1-like (Maldonado *et al.*, 2002; Champigny *et al.*, 2013), as well as nitric oxide (NO) and ROS (Wang *et al.*, 2014), have been implicated in long-distance SAR signalling. An increasing body of evidence suggests that some of these signals interact to coordinate SAR (Dempsey and Klessig, 2012; Shah and Zeier, 2013; Gao *et al.*, 2014; Shah *et al.*, 2014). DIR1 and AZI1, for example, physically interact and might act upstream of G3P accumulation, while G3P in turn appears to stabilize *DIR1* and *AZI1* transcripts and to act together with DIR1 to elicit SAR (Chaturvedi *et al.*, 2008; Yu *et al.*, 2013; Shah *et al.*, 2014). AzA is thought to act upstream of the G3P–*DIR1/AZI1* positive feedback loop (Yu *et al.*, 2013), and NO and ROS were recently placed upstream of AzA in an SAR signalling pathway that appears to act in parallel with SA (Wang *et al.*, 2014).

ENHANCED DISEASE SUSCEPTIBILITY1 (EDS1), together with its sequence-related partners PHYTOALEXIN-DEFICIENT4 (PAD4) and SENESCENCE-ASSOCIATED GENE 101 (SAG101), is an important regulator of SA accumulation, as part of a feedback loop fortifying SA signalling (Falk *et al.*, 1999; Feys *et al.*, 2005; Vlot *et al.*, 2009; Rietz *et al.*, 2011). EDS1 contains a non-catalytic lipase-like domain with a classical α/β hydrolase-fold at its N-terminus and is essential for basal resistance to virulent pathogens as well as ETI mediated by TNL receptors and at least one CNL receptor (Aarts *et al.*, 1998; Zhu *et al.*, 2011; Wagner *et al.*, 2013). EDS1 forms separate nucleocytoplasmic and nuclear heterodimers, respectively, with PAD4 and SAG101 (Feys *et al.*, 2005; Wagner *et al.*, 2013). EDS1 shuttles between the cytoplasm and nucleus via the nuclear pore machinery, and evidence suggests that both its nuclear and cytoplasmic pools contribute to defence (Garcia *et al.*, 2010). Nuclear EDS1 accumulation is essential for TNL-mediated resistance and transcriptional activation of defence genes in ETI (Garcia, 2010). Moreover, EDS1 has been found in nuclear complexes with several TNL receptors as well as their recognized pathogen effectors, suggesting that EDS1 molecularly connects effector recognition to transcriptional defence reprogramming (Bhattacharjee *et al.*, 2011; Heidrich *et al.*, 2011; Zhu *et al.*, 2011; Kim *et al.*, 2012).

**Fig. 1.**
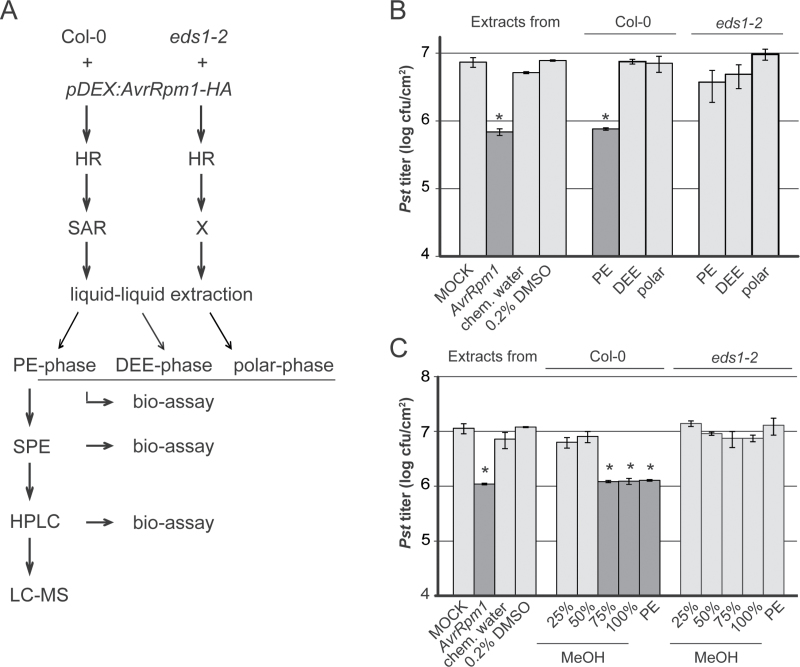
Extraction of SAR-related metabolites from pathogen-free SAR-induced plants. (A) Workflow. Metabolites were extracted with methanol (MeOH) from dexamethasone (DEX)-treated *pDEX:AvrRpm1-HA* Col-0 wild-type (wt) and *pDEX:AvrRpm1-HA eds1-2* mutant plants. Metabolites were purified with a bioassay-assisted approach, including liquid–liquid extraction with petroleum ether (PE) and diethylether (DEE), solid-phase extraction (SPE), and high-performance liquid chromatography (HPLC) coupled with mass spectrometry (MS). (B) SAR bioassay after liquid–liquid extraction. Col-0 plants were locally treated with 10mM MgCl_2_ (MOCK), *Pst*/*AvrRpm1* (*AvrRpm1*), chemical-treated water (chem. water), or 0.2% DMSO as controls or with metabolites from the PE, DEE, or polar phases (as indicated below the panel) derived from Col-0 or *eds1-2* mutant plants (as indicated above the panel). Three days later, systemic leaves were infected with *Pst* and the resulting *Pst* titres are shown 4 d after infection (dpi). Plotted values are the average ±SD from two biologically independent experiments consisting of two replicates each. (C) SAR bioassay after SPE. Col-0 plants were locally treated with the same controls as in (B) or with metabolites from different SPE eluates as indicated below the panel derived from Col-0 or *eds1-2* mutant plants as indicated above the panel. Three days later, systemic leaves were infected with *Pst* and the resulting *Pst* titres are shown at 4 dpi. Plotted values are the average ±SD of three replicates each. (B, C) Asterisks above the bars indicate statistically significant differences from the MOCK or 0.2% DMSO controls (*P<0.05, Student’s *t*-test). These experiments were repeated three times with similar results. HR, hypersensitive response; SAR, systemic acquired resistance.

In resistance mediated by certain CNL receptors, EDS1 acts redundantly with SA (Bartsch *et al*., 2006; Venugopal *et al.*, 2009; Roberts *et al.*, 2013). SA-independent signalling roles of EDS1 have, for example, been associated with responses to the CNLs RPM1 (Bartsch *et al*., 2006) and HRT, recognizing the *Turnip crinkle virus* coat protein (Venugopal *et al.*, 2009), and include a central role in the regulation of SAR (Truman *et al.*, 2007; Rietz *et al.*, 2011; Breitenbach *et al.*, 2014). Both EDS1 and PAD4 are essential for SAR but not local ETI responses to the CNL receptors RPM1 and RPS2 (Aarts *et al.*, 1998; Truman *et al.*, 2007; Jing *et al.*, 2011, Rietz *et al.*, 2011). Recent analysis showed that *EDS1* is necessary both for SAR signal generation in the locally infected tissue and for SAR signal perception in the systemic tissue in RPM1 resistance to *Pseudomonas syringae* expressing the effector AvrRpm1 (Breitenbach *et al.*, 2014). Here, the SAR-specific phenotype of the *eds1* mutant in response to AvrRpm1 was utilized to identify metabolites that are specifically associated with SAR (Fig. 1A). It is reported that the SAR defect of the *eds1* mutant is in part due to a decreased ability to accumulate AzA and its precursors 9-hydroperoxy octadecadienoic acid (9-HPOD) and 9-oxo nonanoic acid (ONA). Application of exogenous ONA and AzA but not the AzA fragmentation product pimelic acid (PIM) induces systemic resistance in *Arabidopsis*. The data reinforce the close association between ONA, AzA, and SAR, and suggest that EDS1 influences the accumulation rate of immune-related lipid peroxidation precursors or products.

## Materials and methods

### Plant material and growth conditions

All experiments were performed in *A. thaliana* ecotype Columbia-0 (Col-0). Mutants *eds1-2*, *npr1-1*, *azi1-2*, *gly1-3*, and *sid2-1* as well as transgenic plants expressing haemagglutinin (HA)-tagged AvrRpm1 from a dexamethasone (DEX)-inducible transgene (*pDEX:AvrRpm1-HA*) in Col-0 and *eds1-2* backgrounds were previously described (Cao *et al.*, 1997; Wildermuth *et al.*, 2001; Mackey *et al.*, 2002; Kachroo *et al.*, 2004; Bartsch *et al*., 2006; Jung *et al.*, 2009; Breitenbach *et al.*, 2014). Plants were grown on normal potting soil mixed with silica sand (ratio 5:1) in 10h light, 14h dark cycles at 70% relative humidity, 22 °C during the day at a light intensity of 100 μE m^–2^ s^–1^, and 18 °C during the night.

### SAR bioassay

All infection experiments were performed in 4- to 5-week-old plants. *Pseudomonas syringae* pathovar *tomato* (*Pst*) and *Pst/AvrRpm1* were maintained as described (Aarts *et al.*, 1998). SAR was induced with *Pst/AvrRpm1* and analysed with a secondary *Pst* infection as described (Breitenbach *et al.*, 2014).

### Metabolite isolation

Lawns of 3- to 4-week-old *pDex:AvrRpm1-HA* plants were sprayed with 30 μM DEX (Sigma Aldrich) dissolved in 0.01% Tween-20. Four to five hours later, 3g of above-ground tissue were harvested per sample and ground in liquid nitrogen. A 30ml aliquot of 100% methanol (MeOH; Merck) was added per sample, and samples were incubated for 1h in the dark while rotating at 28rpm at room temperature. Subsequently, samples were centrifuged at 2800 *g* at 4 °C for 10min and dried by evaporation. Pellets were dissolved in 10ml of MeOH:water (1:9 v/v) and extracted with an equal volume of petroleum ether (PE; Merck). The remaining material was extracted with an equal volume of diethyl ether (DEE, Merck). Both PE and DEE phases were dried by evaporation, and the dry matter was dissolved in 100 μl of dimethylsulphoxide (DMSO; Roth, Germany).

For subsequent solid-phase extraction (SPE), the PE phase in DMSO was diluted with 900 μl of MeOH:water (1:1 v/v). The sample was loaded onto a C18 cartridge (Agilent Technologies, 100mg bed mass, 1ml volume), which was consecutively washed with 5ml of 25, 50, 75, and 100% of MeOH followed by a wash with 5ml of PE. For further fractionation by high-performance liquid chromatography (HPLC), the 75% and 100% MeOH wash eluates and the final PE eluate were pooled, dried by evaporation, and the dry matter was dissolved in 600 μl of MeOH. Finally, the samples were centrifuged at the maximum speed (depending on the rotor) for 15min at 4 °C and the supernatant was used for HPLC.

### Preparative RP18-HPLC-UV/ESI-MS^n^

Preparative HPLC was performed on a Jasco HPLC system (Jasco GmbH, Germany) consisting of two Jasco PU-2087 Plus pumps connected to a Jasco UV-2075 Plus variable wavelength detector set at 260nm, an Advantec CHF122SC fraction collector (Tokyo Seisakusho Kaisha Ltd, Japan), and an Agilent LC/MSD Trap XCT mass spectrometer. The (HP)LC column was a Synergi 4u Fusion-RP 80, 25 cm×21.5mm (Phenomenex). The HPLC solvents were 0.1% formic acid in water (A) and 0.1% formic acid in MeOH (B). For separation of compounds dissolved in 100% MeOH, a gradient was used from 100% A for 2min, then to 100% B in 28min, 20min at these conditions, returning to 100% A at a flow rate of 9.5ml min^–1^. The injection volume was 950 μl per HPLC run. Fractions (9.5ml) were collected at one fraction per minute. Data analysis was performed using the ChromPass Version 1.9.302.1124 software (Jasco GmbH, Germany).

### Analytical LC-MS

A Bruker Daltonics esquire 3000^plus^ ion trap mass spectrometer connected to an Agilent 1100 HPLC system equipped with a quaternary pump and a diode array detector was utilized. Components were separated with a Phenomenex Luna C-18 column (150mm long 2.0mm, particle size 5 μm) held at 28 °C. The injection volume was 5 μl. HPLC was performed with the following binary gradient system: solvent A, water with 0.1% formic acid; and solvent B, 100% MeOH with 0.1% formic acid. The gradient program was as follows: 0–30min, 100% A to 50% A/50% B; 30–35min, 50% A/50% B to 100% B, hold for 15min; 100% B to 100% A, in 5min, then hold for 10min. The flow rate was 0.2ml min^−1^. The full-scan mass spectra were measured in a mass-to-charge (*m/z*) scan range from 50 to 800 with a scan resolution of 13 000 *m/z* s^–1^ until the ICC target reached 20 000ms or 200ms, whichever was achieved first. The ionization parameters were as follows: the voltage of the capillary was 4000V and the end plate was set to –500V. The capillary exit was 121V and the Octopole RF amplitude 150 Vpp. The temperature of the dry gas (N_2_) was 330 °C at a flow of 9 litres min^–1^. Tandem mass spectrometry (MS) was carried out using helium as the collision gas (3.56×10^−6^ mbar) with 1V collision voltage. Auto-tandem MS was used to break down the most abundant [M-H]^–^ or [M+HCOO]^–^ ions of the different compounds. Metabolites were identified by their retention times, mass spectra, and product ion spectra compared with data of authentic reference materials. Data analysis was performed using DataAnalysis 3.1 (Bruker Daltonics).

### 12-Tesla FT-ICR-MS

Ultra-high resolution mass spectra were acquired using a Fourier transform ion cyclotron resonance (FT-ICR) mass spectrometer (Solarix, Bruker) with a 12 Tesla superconducting magnet (Magnex Scientific Varian Inc.). Samples dissolved in 70% MeOH were ionized by electrospray ionization (ESI, Apollo II; Bruker Daltonics) at a flow rate of 2 μl min^–1^. The temperature of the dry gas (N_2_) was 200 °C at a flow of 2 litres min^–1^. Mass spectra were recorded in a scan range of 128–1000 *m/z* with an ion accumulation time of 300ms. A total of 300 scans were accumulated for each MS acquisition. The FT-ICR-MS spectra were normalized by using the exact masses of known plant metabolites including C16 and C18 fatty acids with the Bruker Daltonics data analysis software. For linearization, absolute signal intensities were divided by the maximum amplitude of noise, yielding signal-to-noise (S/N) ratios.

### Chemicals

AzA (Sigma Aldrich), ONA (Chrion AS, Norway), and PIM (Roth, Germany) were each dissolved in MeOH and kept at –80 ºC for a maximum period of 3 months.

### Chemical SAR induction

The first two true leaves of 4- to 5-week-old plants were syringe-infiltrated with the appropriate concentration of a chemical compound or with fractions derived from plant extracts. Three days later, the next two ‘upper’ or systemic leaves were infiltrated with 10^5^ cfu ml^–1^ of *Pst*. Resulting *Pst* titres were determined at 4 d post-infiltration (dpi) as described (Breitenbach *et al.*, 2014). Primary treatments of plants with 0.1% MeOH, 0.2% DMSO, or chemical-treated water were included as negative controls. Chemical-treated water was generated by mixing equal volumes of PE, DEE, MeOH, and water, and evaporating the mixture to remove PE, DEE, and MeOH.

### Cell death assay

Cell death was visualized by Trypan blue staining as described (Aarts *et al.*, 1998) and observed under a light microscope (Olympus BX61).

### RNA isolation and qRT–PCR

Total RNA was isolated using TRI-reagent (Sigma Aldrich) according to the manufacturer’s instructions. cDNA was generated using SuperscriptII reverse transcriptase (Invitrogen). Quantitative PCR (qPCR) was performed using the primers 5′CTACGCAGAACAACTAAGAGGCAAC3′ and 5′TTGGCACA TCCGAGTCTCACTG3′ for *PATHOGENESIS-RELATED1* (*PR1*) and 5′GTACCTTGAAGCTTGCTAATCCTA3′ and 5′GTC AAAGGTGCAAAACCAAC3’ for *TUBULIN* (*TUB*) with the Sensimix SYBR low-rox kit (Bioline) on a 7500 real-time PCR system (Applied Biosystems). Transcript accumulation was analysed using relative quantification with the 7500 Fast System Software 1.3.1. Presented qPCR results are the average of three technical repetitions per sample ± the standard deviation.

## Results

### *EDS1*-dependent SAR is associated with apolar metabolites

It was previously shown that DEX treatment of *pDEX:AvrRpm1-HA* Col-0 wild type (wt) plants (Mackey *et al.*, 2002) induces expression of *AvrRpm1-HA* in the treated leaves and *EDS1*-dependent SAR-like immunity in systemic *AvrRpm1-HA*-non-expressing leaves (Breitenbach *et al.*, 2014; Fig. 1A). The leaves of *pDEX:AvrRpm1-HA* plants emitted SAR signals between 4h and 6h after DEX treatment (Breitenbach *et al.*, 2014). Therefore, metabolite profiles in the above-ground tissue of DEX-treated *pDEX:AvrRpm1-HA* wt and *eds1-2* mutant plants harvested at 4–5h after the DEX treatment were compared. First, metabolites were extracted in MeOH and separated into apolar and polar fractions by liquid–liquid extraction using PE followed by DEE (Fig. 1A). Metabolites in the PE and DEE phases were dried by evaporation and dissolved in DMSO. To allow *in planta* analysis of the SAR-inducing capacity of the metabolites, solutions were diluted with water to a final concentration of 0.2% DMSO. Additionally, PE or DEE remnants were removed from the remaining polar phase.

The SAR-inducing capacity of the different phases isolated from wt and *eds1-2* mutant plants was tested after their infiltration into the first two true leaves of Col-0 wt recipient plants. As a positive control, plants were treated with *Pst/AvrRpm1*. As negative controls, plants were treated with 10mM MgCl_2_ (mock), 0.2% DMSO, or water treated with the chemicals used for liquid–liquid extraction. Three days later, SAR was measured by a challenge infection of the next two upper or systemic leaves of the treated plants with virulent *Pst* and quantification of the resulting *Pst* titres at 4 dpi. Primary treatment of plants with *Pst/AvrRpm1* induced SAR, as indicated by reduced *Pst* titres in the systemic challenge-infected leaves compared with those in the mock-treated control plants (Fig. 1B). A similar degree of systemic resistance was observed in plants that were locally treated with the PE phase derived from DEX-treated *pDEX:AvrRpm1-HA* wt plants compared with the 0.2% DMSO- and chemical-treated water controls (Fig. 1B). In contrast, the DEE and polar phases from wt plants did not induce SAR. Similarly, the PE, DEE, or polar phases from *eds1-2* mutant plants failed to induce SAR in wt plants (Fig. 1B). Thus, non-polar, PE-soluble SAR-inducing metabolites accumulated in extracts from DEX-treated *pDEX:AvrRpm1-HA* plants in an *EDS1*-dependent manner.

In the next purification step, metabolites contained in the PE phases from DEX-treated *pDEX:AvrRpm1-HA* wt and *eds1-2* mutant plants were fractionated by SPE (Fig. 1A). C18 columns were loaded with the respective PE phases and consecutively washed with 25, 50, 75, and 100% MeOH followed by a final PE wash. Each wash eluate was dried by evaporation and dissolved in DMSO. Subsequently, the eluates were diluted with water to 0.2% DMSO and infiltrated into the first two true leaves of Col-0 plants. At 3 dpi, systemic leaves were challenged with *Pst* and the resulting *Pst* titres were determined at 4 dpi. Compounds derived from wt plants and eluting from C18 columns in 75% and 100% MeOH or in PE induced SAR (Fig. 1C). Compared with the respective negative control treatments, these eluates induced a similar reduction of *Pst* titres in the systemic challenge-infected tissue as the *Pst/AvrRpm1*-positive control treatment. In contrast, compounds eluting in 25% or 50% MeOH did not elicit SAR. Similarly, SPE eluates derived from *eds1-2* mutant plants did not induce SAR (Fig. 1C). Together, these results confirmed the non-polar nature of *EDS1*-dependent SAR signalling components. In addition to a reduced capacity to accumulate apolar SAR-inducing compounds, the *eds1-2* mutant also did not support systemic resistance in response to the SAR-inducing fractions derived from wt plant extracts (Supplementary Fig. S1 available at *JXB* online).

### HPLC-assisted fractionation of SAR-inducing activities

**Fig. 2.**
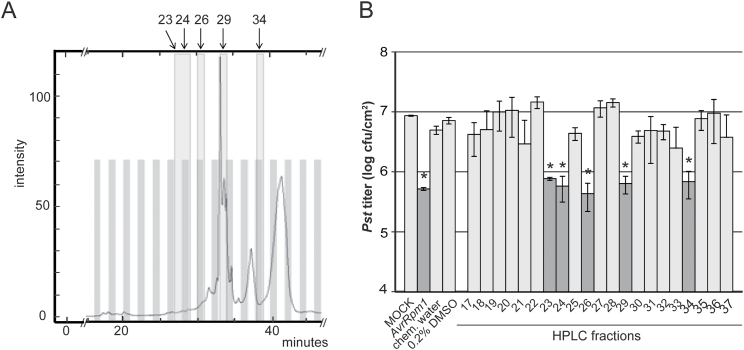
HPLC-assisted separation of SAR-inducing metabolites. (A) UV absorption signal of an MeOH gradient HPLC chromatogram derived from DEX-treated *pDEX:AvrRpm1-HA* Col-0 plants. The signal intensity at 260nm (y-axis) is shown against the HPLC retention time in minutes (*x*-axis). One fraction was collected per minute and fractions 17–37 (analysed in B) are shown as alternating grey and white bars. SAR-inducing fractions are further highlighted in light grey and numbered above the panel. (B) SAR bioassay of HPLC fractions 17–37. Col-0 plants were locally treated with 10mM MgCl_2_ (MOCK), *Pst/AvrRpm1* (*AvrRpm1*), chemical-treated water (chem. water), or 0.2% DMSO as controls or with HPLC fractions 17–37 derived from wt plants (A) in 0.2% DMSO. Three days later, systemic leaves were infected with *Pst* and the resulting *Pst* titres are shown 4 d after infection (dpi). Plotted values are the average ±SD of three replicates each. Asterisks above the bars indicate statistically significant differences from the MOCK or 0.2% DMSO controls (*P<0.05, Student’s *t*-test). This experiment was repeated three times with comparable results.

For HPLC, the 75% and 100% MeOH and final PE SPE eluates from 10–20 biologically independent extractions were pooled per plant genotype, dried by evaporation, and dissolved in MeOH. Thus, compounds derived from 30–60g of plant material per genotype were separated across a MeOH gradient in 10–20 consecutive preparative HPLC runs. During each run, 40 fractions were collected of 9–10ml each and the corresponding fractions of consecutive runs were pooled (Fig. 2A; Supplementary Fig. S2A at *JXB* online). Fractions 17–37 (corresponding to 75–100% MeOH) were dried by evaporation. For SAR assays, the solid matter in each fraction was dissolved in 200–300 μl of DMSO, diluted with water to 0.2% DMSO, and infiltrated into the first two true leaves of wt plants. SAR was then analysed as described above. In the experiment shown in Fig. 2B, primary treatments of plants with HPLC fractions 23, 24, 26, 29, and 34 derived from wt plants induced SAR, causing a reduction of *Pst* titres in systemic challenge-infected tissue to the same level as the positive control primary treatment with *Pst/AvrRpm1*. The corresponding HPLC fractions from *eds1-2* mutant plants did not induce SAR (Supplementary Fig. S2B), providing further evidence that the HPLC-separable SAR-inducing activities derived from the wt plants are *EDS1* dependent. Reciprocally, the SAR-inducing fractions derived from wt plants or the corresponding fractions derived from *eds1-2* mutant plants did not induce SAR in *eds1-2* mutant plants (Supplementary Fig. S2C), confirming that the *eds1-2* mutant also does not respond to *EDS1*-dependent SAR signals derived from wt plants (Breitenbach *et al.*, 2014).

### MS-assisted identification of SAR-related metabolites

**Fig. 3.**
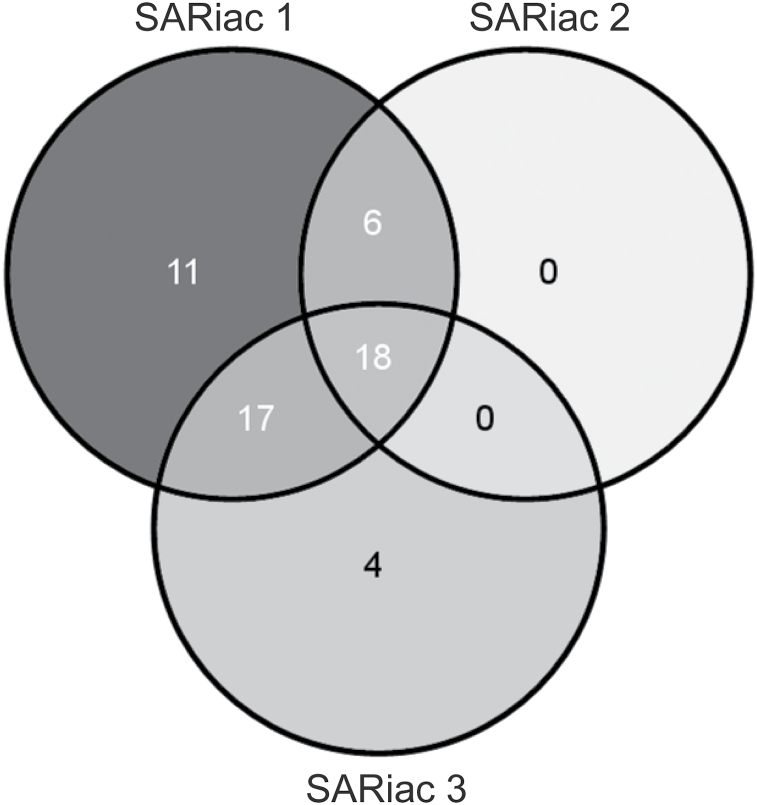
Venn diagram of annotated metabolites accumulating in SAR-inducing activity (SARiac) 1–3 in an *EDS1-*dependent manner as detected by FT-ICR-MS (Oliveros, 2007). This experiment was repeated twice with similar results.

Because the SAR-inducing activity contained in HPLC fractions 23, 24, and 26 sometimes resolved in a single or two HPLC fractions, the SAR-inducing fractions from this range of the HPLC chromatogram were pooled and defined as ‘SAR-inducing activity 1’ (SARiac 1). Fractions 29 and 34 were analysed as SARiac 2 and 3, respectively. First, FT-ICR-MS was used to analyse negatively charged [M-H]^–^ ions in SARiac 1–3 from wt plants compared with the corresponding HPLC fractions from the *eds1-2* mutant. Mass spectra were acquired in the negative ionization mode focusing on organic compounds that bear hydroxyl or carboxyl groups and can be easily dissolved in MeOH. The generated mass spectra were normalized and the signal intensities converted to a linear S/N ratio scale (see the Materials and methods). Subsequently, masses were selected that accumulated in SARiac 1–3 in an *EDS1*-dependent manner if their S/N ratio was at least 5-fold higher in the fractions derived from wt plants compared with corresponding fractions from the *eds1-2* mutant. The selected masses were queried against the KEGG, Knapsack, and Human Metabolome DataBase for annotation (Wishart *et al.*, 2007; Afendi *et al.*, 2012; Kanehisa *et al.*, 2014). As a result, 56 annotated masses were found (Supplementary Table S1 at *JXB* online). Of these, 18 metabolites were associated with SARiac 1–3 (Fig. 3; Table 1). Notably, all of the *EDS1*-dependent metabolites identified in SARiac 2 were shared with SARiac 1. This result suggests that the separation of compounds by preparative HPLC was suboptimal. Nevertheless, 56 identified metabolites accumulated in SAR-inducing HPLC fractions from DEX-treated *pDEX:AvrRpm1-HA* plants in an *EDS1*-dependent manner and therefore are potentially associated with systemic immunity, possibly acting in concert and/or in a concentration-dependent manner.

**Table 1.** Putative SAR-related metabolites that are shared between SARiac 1, 2, and 3The identification number (ID) corresponds to numbering in Supplementary Table S1 at *JXB* online. This experiment was repeated twice with similar results.

ID	Theoretical mass [M-H]^–^	Experimental mass [M-H]^–^	Annotated as	Chemical formula
3	243.066285	243.066307	3,3′,4′5-Tetrahydroxystilbene	C14H12O4
7	269.04555	269.045579	Sulphuretin	C15H10O5
17	315.087415	315.087402	Cajanol	C17H16O6
20	405.11911	405.119227	Astringin	C20H22O9
21	415.10346	415.103598	Daidzin	C21H20O9
24	421.114025	421.114185	Plicatic acid	C20H22O10
26	431.098375	431.098472	Vitexin	C21H20O10
27	431.13476	431.134868	2-(2,4,5-Trimethoxyphenyl)-5,6,7,8-tetramethoxy-4H-1-benzopyran-4-one	C22H24O9
28	433.114025	433.114164	Phlorizin chalcone	C21H22O10
29	435.09329	435.093389	Irisxanthone	C20H20O11
32	445.114025	445.114141	Biochanin A-β-d-glucoside	C22H22O10
36	449.10894	449.109062	2′,3,4,4′,6′-Peptahydroxychalcone 4′-*O*-glucoside	C21H22O11
38	461.10894	461.109081	Isoscoparine	C22H22O11
43	477.103855	477.103989	Isorhamnetin 3-*O*-β-d-glucopyranoside	C22H22O12
46	491.119505	491.119659	Aurantio-obtusin β-d-glucoside	C23H24O12
49	519.18719	519.18737	Brusatol	C26H32O11
54	563.140635	563.141039	Apigenin 7-*O*-[β-d-apiosyl-(1→2)-β-D-glucoside]	C26H28O14
56	609.146115	609.146474	Lucenin-2	C27H30O16

**Fig. 4.**
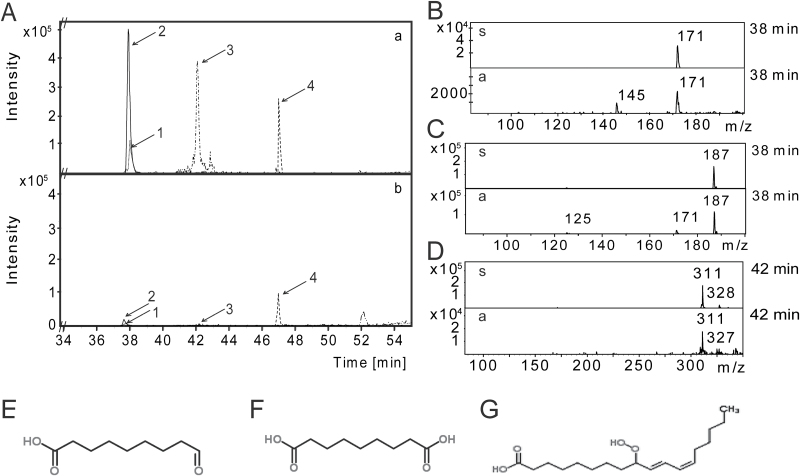
LC-MS analysis of SARiac 1 from DEX-treated *pDEX:AvrRpm1-HA* Col-0 plants and the corresponding fractions from DEX-treated *pDEX:AvrRpm1-HA eds1-2* mutant plants. (A) Intensity peaks (*y*-axis) detected in the negative ionization mode and their LC retention time in minutes (*x*-axis) of masses that differentially accumulated in extracts from Col-0 (upper panel, a) and *eds1-2* (lower panel, b) plants. (1) ONA, 9-oxo nonanoic acid; (2) AzA, azelaic acid; (3) 9-HPOD, 9-hydroperoxy octadecadienoic acid; and (4) an unknown compound. (B–D) LC-MS of metabolites 1–3 in fractions derived from wt plants (bottom half of each panel) compared with the respective ONA (B), AzA (C), and 9-HPOD (D) standards (upper half of each panel). Mass-to-charge (*m/z*) ratios are indicated above each peak and LC retention times in minutes to the right of each panel. (E–G) Chemical structures of ONA (E), AzA (F), and 9-HPOD (G) from www.chemspider.com (last accessed July 2014). This experiment was repeated twice with similar results.

Because an SARiac-associated mass was detected by FT-ICR-MS that might correspond to the putative SAR signal AzA at a signal intensity close to the background noise, SARiac 1 was analysed further by liquid chromatography (LC) coupled with ion trap MS/MS (see the Materials and methods). Using this method, four masses were detected that were predominantly present in SARiac 1 from wt plants compared with corresponding fraction(s) from the *eds1-2* mutant (Fig. 4A). By comparing the LC retention times, MS, and MS^2^ data with different standards, the peaks with pseudo-molecular ions at *m/z* 171, 187, and 311 were identified as ONA (Fig. 4B, E), AzA (Fig. 4C, F), and 9-HPOD (Fig. 4D, G), respectively. It was not possible to identify the fourth EDS1-dependent metabolite showing a pseudo-molecular ion at *m/z* 255. In contrast to the 56 metabolites identified by FT-ICR-MS, ONA, AzA, and 9-HPOD were found to be relatively unstable during storage of the samples. SARiac 1, for example, typically lost ONA and AzA and much of the 9-HPOD after 3 months of storage at –80 °C. Notably, this was associated with a loss of SAR-inducing activity. Initial evidence suggested that the SAR-inducing activity of SARiac 2 was similarly related to *EDS1*-dependent accumulation of ONA and AzA, but not 9-HPOD (Supplementary Fig. S3 at *JXB* online). Together, the data relate the SAR defect of *eds1* mutant plants with reduced accumulation of ONA and AzA.

### Exogenous ONA induces SAR more efficiently than exogenous AzA

**Fig. 5.**
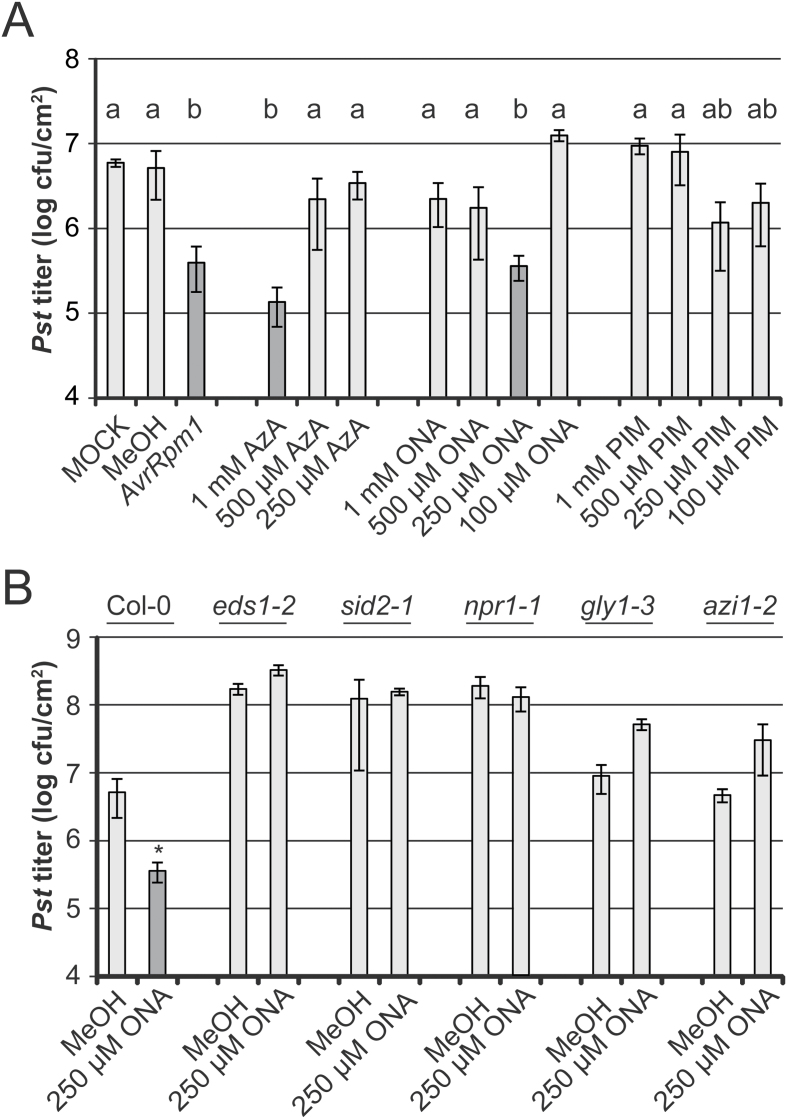
Induction of systemic resistance by ONA, AzA, and PIM application. (A) SAR bioassay in wt plants. Col-0 plants were locally treated with 10mM MgCl_2_ (MOCK), 0.1% MeOH, *Pst/AvrRpm1* (*AvrRpm1*), or with different concentrations of AzA, ONA, or PIM as indicated below the panel. Three days later, systemic leaves were infected with *Pst* and the resulting *Pst* titres are shown at 4 dpi. Plotted values are the average ±SD of three replicates each. Results marked with different letters above the bars are statistically different (*P*<0.05, Student’s *t*-test). (B) ONA-induced SAR in different mutants. Col-0 plants or the mutants indicated above the panel were locally treated with 0.1% MeOH or 250 μM ONA as indicated below the panel. SAR was analysed as in (A). An asterisk above the bar indicates a statistically significant difference from the 0.1% MeOH control (*P<0.05, Student’s *t*-test). These experiments were repeated at least three times with similar results.

9-HPOD can be fragmented to yield ONA, and exogenous ONA is readily oxidized to AzA in *Arabidopsis* (Fig. 7; Zoeller *et al.*, 2012; Farmer and Mueller, 2013; Yu *et al.*, 2013; Wang *et al.*, 2014). Additionally, it has been reported that exogenous AzA in *Arabidopsis* is converted within 24h into the C7 dicarboxylic acid PIM (Zoeller *et al.*, 2012). Here, the SAR-inducing capacity of exogenously applied ONA, AzA, and PIM, was tested, but that of 9-HPOD could not be tested because a reasonable concentration of 9-HPOD in water could not be obtained for plant treatments. The first two true leaves of Col-0 plants were treated with different concentrations of ONA, AzA, or PIM. Alternatively, plants were treated with *Pst/AvrRpm1* as a positive control or with 10mM MgCl_2_ or 0.1% MeOH as negative controls. SAR was analysed as above with a systemic *Pst* challenge infection. As previously observed (Jung *et al.*, 2009; Chaturvedi *et al.*, 2012), primary treatment of plants with 1mM AzA induced systemic resistance, causing a reduction in systemic *Pst* titres to a similar degree as the *Pst/AvrRpm1* positive control primary treatment (Fig. 5A). Application of lower concentrations of AzA did not elicit SAR. Primary treatment of plants with 250 μM ONA induced systemic resistance to *Pst*, whereas the application of higher or lower concentrations of ONA did not (Fig. 5A). PIM did not trigger significant SAR when applied at the concentrations tested, although primary treatments of plants with 250 μM or 100 μM PIM induced an SAR trend that was not statistically different from the positive or negative controls (Fig. 5A). Taken together, the data show that application of ONA and AzA but not PIM induces SAR.

It is currently unclear why ONA did not trigger systemic resistance when applied at 1mM or 500 μM. Because SAR is often but not always associated with primary treatments that induce cell death (Cameron *et al.*, 1994; Durrant and Dong, 2004; Mishina and Zeier, 2007; Liu *et al.*, 2010), it was investigated whether the application of different concentrations of ONA or AzA induced different degrees of cell death by staining the treated leaves with Trypan blue (Supplementary Fig. S4 at *JXB* online). In contrast to the positive control treatment with *Pst/AvrRpm1*, which induced cell death, ONA and AzA treatments did not trigger more cell death than the negative control treatments with 10mM MgCl_2_ or 0.1% MeOH at any ONA and AzA concentration tested (Supplementary Fig. S4). Thus, SAR induced by ONA or AzA application does not appear to be associated with localized cell death, although it cannot be excluded that the accumulation of ONA or AzA during biologically induced SAR might be. Subsequently, the integrity of the commercial ONA used, which was kept in MeOH at –80 °C, was tested. After 3 months of storage, ~16% of ONA was oxidized to AzA, as determined by LC-MS (Supplementary Fig. S5A, B). Infiltration of this mixture into Col-0 leaves caused a rapid further oxidation of ONA within 4h post-infiltration (hpi), supporting previous findings using isotope-labelled ONA that ONA is readily converted to AzA in planta (Zoeller *et al.*, 2012; Supplementary Fig. S5C). However, the possibility that exogenous ONA induced SAR independently of its *in planta* oxidation cannot be excluded, because ONA remained detectable and elevated compared with its basal level in leaf extracts until 72h after infiltration of leaves with 250 μM ONA (Supplementary Fig. S5C). Alternatively, exogenous ONA may be more membrane permeable than AzA and thus induce SAR via its oxidation, producing similar intracellular AzA accumulation when applied at ~250 μM (Supplementary Fig. S5A) compared with AzA applied at 1mM.

It was next investigated whether ONA contributes to SAR via a similar mechanism to AzA. As *eds1-2*, the SA biosynthesis mutant *sid2-1* and the SA signalling mutant *npr1-1* display enhanced susceptibility to *Pst* and are SAR defective (Fig. 5B; Cao *et al.*, 1997; Wildermuth *et al.*, 2001). Treatment of these and *pad4* mutant plants with AzA did not enhance resistance against *Pst*, indicating that AzA acts upstream of SA (Jung *et al.*, 2009). In the assays performed here, *eds1-2*, *sid2-1*, and *npr1-1* mutant plants also failed to induce SAR in response to applications of 250 μM ONA (Fig. 5B). AzA-induced resistance was found to be dependent on G3P and AZI1 (Jung *et al.*, 2009; Yu *et al.*, 2013). For comparison, it was tested whether ONA application elicits systemic resistance in the *gly1-3* mutant, which is compromised for G3P accumulation and SAR (Chanda *et al.*, 2011), or in *azi1-2* mutant plants. Both mutants displayed normal (wt-like) susceptibility to *Pst*, but did not support SAR in response to the application of 250 μM ONA (Fig. 5B). Taken together, these results suggest that the mechanisms leading to SAR downstream of ONA and AzA application are related since they are dependent on *EDS1* and/or *PAD4*, SA, *AZI1*, and G3P.

### Responses to AzA depend on the AzA concentration applied

**Fig. 6.**
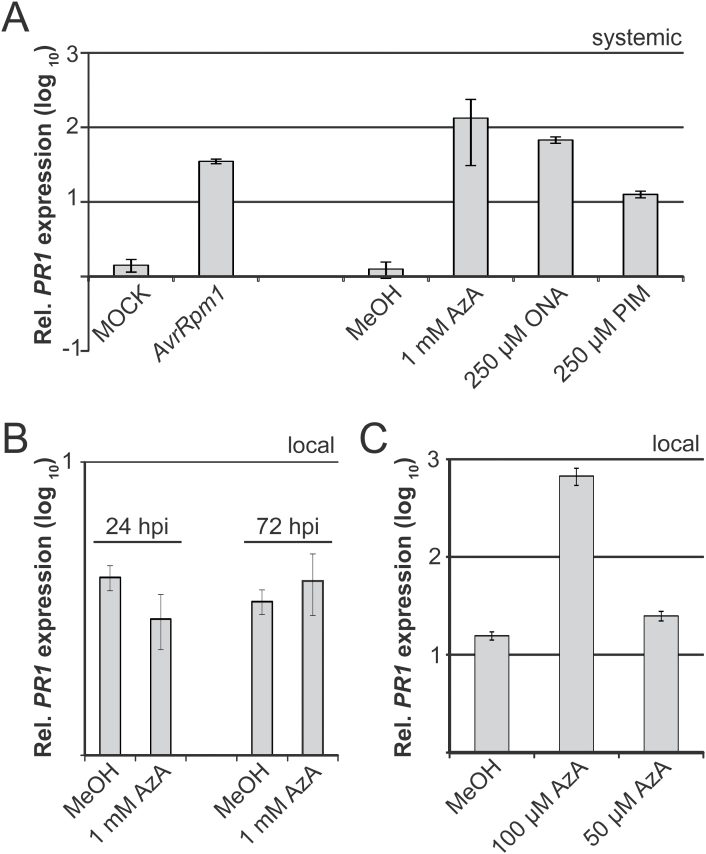
*PR1* transcript accumulation in response to ONA, AzA, or PIM application (A) Systemic *PR1* induction. Plants were locally treated with 10mM MgCl_2_ (MOCK), *Pst/AvrRpm1* (*AvrRpm1*), 0.1% MeOH, 1mM AzA, 250 μM ONA, or 250 μM PIM. Three days later, *PR1* transcript accumulation in systemic untreated leaves was analysed by qRT–PCR and normalized to that of the reference gene *TUBULIN*. The normalized expression is shown relative to that in leaf tissue from untreated Col-0 plants. (B, C) Local *PR1* transcript accumulation in leaves treated with 0.1% MeOH or AzA at different concentrations as indicated below the panels. *PR1* transcript accumulation was analysed as in (A) and samples were taken at the time points indicated in (B) or at 72 hpi (C). These experiments were repeated at least three times with similar results. Rel., relative; hpi, hours post-infiltration

To investigate plant responses to ONA and AzA applications further, the transcript accumulation of the SAR marker gene *PR1* was analysed in systemic untreated leaves at 3 d after a local treatment of the plants with 1mM AzA, 250 μM ONA, or 250 μM PIM, and this was compared with a positive control treatment with *Pst/AvrRpm1* and negative control treatments with 10mM MgCl_2_ or 0.1% MeOH. Similar to the positive control treatment, ONA and AzA application induced *PR1* transcript accumulation in systemic untreated leaves, whereas a local PIM application caused much lower systemic induction of *PR1* transcripts (Fig. 6A). As previously shown (Jung *et al.*, 2009), the same AzA treatment did not enhance local *PR1* transcript accumulation in the treated tissue (Fig. 6B). Because up to 7% of exogenous AzA was reported to move systemically in *Arabidopsis* (Yu *et al.*, 2013), the local response to applications of 50 μM and 100 μM AzA was investigated and it was observed that application of 100 μM but not 50 μM AzA locally induced *PR1* transcript accumulation (Fig. 6C). These results indicate that responses to exogenous AzA depend on the AzA concentration applied and that exogenous AzA might induce SAR after travelling from the local treated site to the systemic site.

## Discussion

The C9 dicarboxylic acid AzA accumulates in infected leaves and petiole exudates of plants infected with *P. syringae* expressing the effector *AvrRpt2* (Jung *et al.*, 2009; Yu *et al.*, 2013). Here, it is shown that AzA accumulates together with its immediate precursor ONA in extracts of *AvrRpm1-HA*-expressing plants in an *EDS1*-dependent manner (Fig. 4). Because SAR signal generation, but not local resistance in response to *AvrRpm1*, is compromised in *eds1* mutant plants, these results associate ONA and AzA specifically with SAR rather than local resistance responses (Aarts *et al.*, 1998; Truman *et al.*, 2007; Rietz *et al.* 2011; Breitenbach *et al.*, 2014). Along with ONA, AzA, and one of their precursors, 9-HPOD, 56 additional annotated metabolites were detected whose accumulation in extracts from *AvrRpm1*-expressing plants depended on EDS1 (Fig. 3; Supplementary Table S1 at *JXB* online). However, ONA and AzA appear to be important for the SAR-inducing activity of fractions from plant extracts because their loss during storage of samples correlated with a loss of SAR-inducing activity. Thus, although other *EDS1*-dependent metabolites may have supportive functions during SAR, the data suggest that the SAR defect of the *eds1* mutant is in part caused by reduced accumulation of ONA and AzA.

Exogenous ONA induced SAR when applied at a 4-fold lower concentration compared with AzA (Fig. 5). The data reinforce previous findings that exogenous ONA is rapidly oxidized to AzA in *Arabidopsis* leaves (Zoeller *et al.*, 2012). Nevertheless, ONA levels remained detectable and above basal levels for at least 72h after its application (Supplementary Fig. S5 at *JXB* online). Therefore, it is possible that ONA application induces SAR by actions that are independent of AzA. In contrast to AzA, ONA does not appear to accumulate in its free form in plants, but might, for example, remain esterified to galactolipids (Zoeller *et al.*, 2012). In this context, the possibility cannot be excluded that C18 hydroperoxides such as 9-HPOD served as a substrate for fragmentation during the extraction procedure to yield ONA and AzA in *AvrRpm1-HA*-induced extracts (Figs 1, 2). In contrast, a small proportion of AzA accumulates in its free form *in planta* (Zoeller *et al.*, 2012). Up to 7% of exogenous ^14^C-labelled AzA could be detected in systemic tissues away from the site of application, and most of the systemic [^14^C]AzA was detected in AzA derivatives (Yu *et al.*, 2013; Gao *et al.*, 2014). Gao *et al.* (2014) questioned the biological significance of AzA mobility in plants and proposed that AzA might enhance systemic resistance via a local function upstream of G3P in the primary infected tissues. In support of this idea, AzA application locally induces transcript accumulation of *AZI1* (Jung *et al.*, 2009; Yu *et al.*, 2013), which is required for SAR signal emission from the primary infected leaves, but not for systemic SAR signal perception (Jung *et al.*, 2009). Also, in preliminary experiments, no induction of *AZI1* was detected in systemic untreated leaves of plants treated locally with ONA or AzA (Supplementary Fig. S6 at *JXB* online). A local signalling function of membrane-tethered ONA would fit well with a putative SAR-specific signalling event in the primary infected tissue that is independent of the systemic mobility of AzA. Similar to exogenous AzA, exogenous ONA also appears to depend on accumulation of the putative mobile SAR signal G3P to induce systemic resistance (Fig. 5; Yu *et al.*, 2013). Thus, G3P might be the mobile compound transferring signalling from locally infected to systemic tissues in response to localized actions of ONA or AzA (Gao *et al.*, 2014). Alternatively, exogenously applied ONA might act in SAR via its oxidation to AzA and could elicit SAR when applied at a lower concentration due to its enhanced membrane permeability compared with AzA. Whereas exogenous AzA might act locally, low levels of AzA moving systemically in the plant could suffice for eliciting systemic responses. This is supported by the local *PR1* induction observed upon application of 100 μM AzA which was comparable with the level of systemic *PR1* induced by a local application of 1mM AzA (Fig. 6).

It was previously proposed that AzA primes immunity by enhancing SA and *PR1* transcript accumulation upon *P. syringae* challenge infection of AzA-treated *Arabidopsis* leaves (Jung *et al.*, 2009). Priming could be detected from 6h until 18h (for SA) or 24h (for *PR1* transcript accumulation) after the challenge infection of the AzA-treated leaves. A second independent study reported a very modest priming effect of AzA on SA and *PR1* transcript accumulation detected at 6h or 12h after challenge infection of AzA-treated tissue (Yu *et al.*, 2013). Notably, neither study reported an induction of *PR1* transcript accumulation in the AzA-treated leaves before the challenge infection. Here, *PR1* transcript accumulation was detected in systemic untreated leaves of plants locally treated with either ONA or AzA (Fig. 6). This induction was similar to that in systemic uninfected leaves of locally *Pst/AvrRpm1*-infected plants. Sometimes a further priming of *PR1* transcript accumulation was detected at 6h after challenge infection of the systemic tissue, but priming was marginal and not always reproducible, and the data were therefore not included here. Application of the AzA fragmentation product PIM was considerably less effective compared with ONA and AzA applications (Figs 5, 6). PIM application moderately induced systemic *PR1* expression, but a significant SAR response was not recorded. Although additional fragmentation products derived from C18 unsaturated fatty acids have been associated with systemic resistance (Vicente *et al.*, 2012), the present data suggest that the lipid peroxidation products ONA and AzA promote SAR associated with the systemic accumulation of *PR1* transcripts but not necessarily priming. Additionally, the sensitivity of immunity-related responses to the concentration of AzA applied might explain the inverse correlation between AzA levels and the extent of SAR discussed by Zoeller *et al.* (2012).

**Fig. 7.**
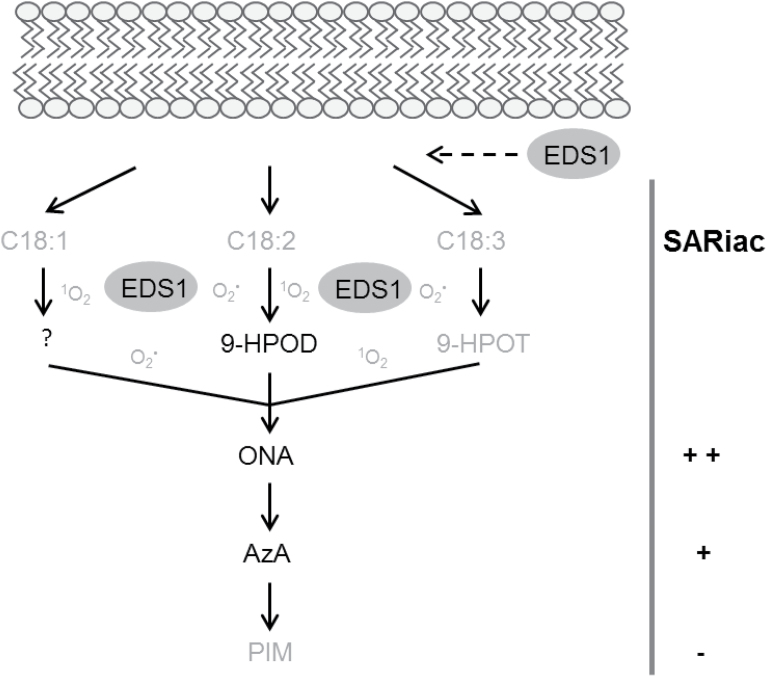
Working model. ONA and AzA are generated by peroxidation of C18 unsaturated fatty acids in an *EDS1-*regulated manner. Compounds identified here as differentially accumulating in extracts from *AvrRpm1-HA-*expressing wt and *eds1* mutant plants are depicted in black; intermediates that were not identified in this study are depicted in grey. EDS1 may directly or indirectly affect the release of C18 unsaturated fatty acids or one or more of their downstream lipid peroxidation products from galactolipid bilayers (striped arrow below the lipid bilayer cartoon). Alternatively, EDS1 may directly or indirectly regulate auto-oxidation of ONA and AzA precursors by regulating ROS homeostasis. The SAR-inducing activity (SARiac) of exogenous ONA, AzA, and PIM is indicated to the right of the cartoon. ^1^O_2_, singlet oxygen, O_2_·^–^, superoxide radical

Transcriptomic and proteomic studies have investigated *EDS1*-dependent responses to *Pst/AvrRpm1* or *AvrRpm1-HA* in order to delineate local and SAR-related events (Bartsch *et al*., 2006; Breitenbach *et al.*, 2014). Until now, three genes identified in these studies have been related to SAR *in planta*. *FLAVIN-DEPENDENT MONOOXYGENASE 1* (*FMO1*) is essential for SAR, acting upstream of SA in the systemic tissue (Mishina and Zeier, 2006). Locally, *FMO1* affects resistance downstream of *EDS1*, in parallel with SA (Bartsch *et al*., 2006). *APOPLASTIC, EDS1-DEPENDENT 1* (*AED1*) and *LEGUME LECTIN-LIKE PROTEIN 1* (*LLP1*) act, negatively and positively, respectively, in SAR with limited effects if any on local resistance responses to different *P. syringae* strains (Armijo *et al.*, 2013; Breitenbach *et al.*, 2014). The data suggest that *LLP1* promotes SAR by acting in parallel with SA (Breitenbach *et al.*, 2014). EDS1 was also found to act redundantly with SA in resistance mediated by the CNL receptor HRT (Venugopal *et al.*, 2009), and a related action might regulate the accumulation of ONA and AzA in response to *AvrRpm1*. Non-enzymatic peroxidation of C18 unsaturated fatty acids is believed to be the main source of AzA *in planta* (Fig. 7; Zoeller *et al.*, 2012; Yu *et al.*, 2013; Wang *et al.*, 2014). The alternative enzymatic route downstream of 9-lipoxygenase (9-LOX) activity was excluded because a double mutant lacking both *Arabidopsis* 9-LOX enzymes accumulated normal AzA levels in response to *Pst/AvrRpm1* (Zoeller *et al.*, 2012). Recent evidence suggests that peroxidation of C18 unsaturated fatty acids is promoted by ROS downstream of NO (Wang *et al.*, 2014). Because NO and ROS trigger systemic resistance via a pathway acting in parallel with SA and upstream of G3P and presumably AzA (Yu *et al.*, 2013; Wang *et al.*, 2014), it is conceivable that SA-independent ROS-driven accumulation of ONA and AzA is promoted by EDS1 in SAR.

An increasing body of evidence suggests that EDS1-mediated signalling affects ROS homeostasis, for example downstream of the non-canonical CNL protein ACTIVATED DISEASE RESISTANCE1 (ADR1; Roberts *et al.*, 2013). ADR1 promotes SA accumulation in resistance mediated by TNL receptors and the CNL receptor RPS2 but not RPM1 (Bonardi *et al.*, 2011). A function of EDS1 acting in parallel with SA regulates signalling downstream of ADR1 and this might be associated with the role of EDS1 in the run-away cell death (RCD) phenotype of the *lesion simulating disease1* (*lsd1*) mutant (Roberts *et al.*, 2013). RCD in *lsd1* mutant plants can be initiated by various biotic and abiotic stresses and is thought to depend on EDS1 promoting H_2_O_2_ accumulation (Rustérucci *et al.*, 2001; Mateo *et al.*, 2004; Mühlenbock *et al.*, 2008; Wituszynska *et al.*, 2013). Similar to SAR, *AvrRpm1*-induced *lsd1* RCD does not appear to be associated with localized *EDS1*-dependent HR-related responses (Rustérucci *et al.*, 2001), suggesting that EDS1 functioning in ROS homeostasis might be associated with SAR. However, H_2_O_2_ is probably not a strong enough radical to support fragmentation of C18 unsaturated fatty acids, which is induced *in vitro* by singlet oxygen (^1^O_2_) and to a lesser extent by superoxide radicals (O_2_·^–^) but not by H_2_O_2_ (Mueller *et al.*, 2006; Farmer and Mueller, 2013; Wang *et al.*, 2014). Available evidence places EDS1 downstream of both ^1^O_2_ and O_2_·^–^ (Ochsenbein *et al.*, 2006; Straus *et al.*, 2010). In the conditional *flu* mutant that hyperaccumulates ^1^O_2_ upon a dark-to-light shift, an *EDS1*-dependent pathway appears to ‘quench’ ^1^O_2_, contributing to recovery of the *flu* mutant from oxidative stress (Ochsenbein *et al.*, 2006). Additionally, Straus *et al.* (2010) provided evidence that EDS1 responds to chloroplast-derived O_2_·^–^ to coordinate SA- and H_2_O_2_-associated cell death and immune signalling. Although spatial separation of different ROS and a possible role of EDS1 upstream of ^1^O_2_ or O_2_·^–^ at specific sites (Fig. 7; Straus *et al.*, 2010) cannot be ruled out, a putative role for EDS1 promoting lipid peroxidation requires further investigation.

Alternatively, EDS1 might affect the release of ONA, AzA, or one or more of their common precursors from galactolipids (Fig. 7) because EDS1 and its partner PAD4 each have a conserved esterase catalytic triad embedded within an α/β-fold hydrolase topology (Falk *et al.*, 1999; Wagner *et al.*, 2013). However, mutation of the predicted catalytic residues of EDS1 and PAD4 did not compromise their functions in ETI or basal resistance responses, and no EDS1 hydrolase activity has so far been detected (Wagner *et al.*, 2013). Taken together, it seems likely that EDS1 indirectly promotes ONA and AzA accumulation in SAR, for example by activating one or more signalling pathways. The nudix hydrolase NUDT7 is induced in *Arabidopsis* by *Pst/AvrRpm1* downstream of *EDS1* and possibly acts in parallel with SA to suppress immune-related cell death associated with ROS (Bartsch *et al*., 2006; Straus *et al.*, 2010). However, the nature of *EDS1*-dependent, possibly SA-independent pathways that promote ONA and AzA accumulation, including a putative role for NUDT7 in SAR, require further investigation.

## Supplementary data

Supplementary data are available at *JXB* online.

Figure S1. SAR bioassays in *eds1-2* mutant plants.

Figure S2. HPLC-assisted separation of SAR-inducing metabolites and their dependency on *EDS1*.

Figure S3. The SAR-inducing activity of SARiac 2 is associated with the accumulation of ONA and AzA.

Figure S4. Trypan blue staining of ONA- and AzA-treated leaves.

Figure S5. LC-MS of ONA after storage at –80 ºC and after infiltration into plants.

Figure S6. Systemic *AZI1* expression in response to local ONA and AzA applications.

Table S1. Annotated metabolites identified by FT-ICR-MS in SARiac 1–3.

Supplementary Data

## Abbreviations:

AzA: azelaic acid
DEE: diethylether
DEX: dexamethasone
DMSO: dimethylsulphoxide
EDS1: ENHANCED DISEASE SUSCEPTIBILITY1
ETI: effector-triggered immunity
FT-ICR: Fourier transform ion cyclotron resonance
G3P: glycerol-3-phosphate
HPLC: high-performance liquid chromatography
9-HPOD: 9-hydroperoxy octadecadienoic acid
HR: hypersensitive response
MeOH: methanol
MS: mass spectrometry
ONA: 9-oxo nonanoic acid
PAMP: pathogen-associated molecular pattern
PE: petroleum ether
PIM: pimelic acid
PTI: PAMP-triggered immunity
*PR1*: *PATHOGENESIS-RELATED1*
*Pst*: *Pseudomonas syringae* pathovar *tomato*
ROS: reactive oxygen species
SA: salicylic acid
SAR: systemic acquired resistance
SPE: solid-phase extraction.
