# Supplementary material for: Arabidopsis ENHANCED DISEASE SUSCEPTIBILITY1 promotes systemic acquired resistance via azelaic acid and its precursor 9-oxo nonanoic acid
Source: J Exp Bot. 2014 Aug 11;65(20):5919–31. doi: 10.1093/jxb/eru331 (PMC4203127; doi:10.1093/jxb/eru331)
Supplement: Supplementary Data [file supp_65_20_5919__index.html]

 Arabidopsis ENHANCED DISEASE SUSCEPTIBILITY1 promotes systemic acquired resistance via azelaic acid and its precursor 9-oxo nonanoic acid — Arabidopsis ENHANCED DISEASE SUSCEPTIBILITY1 promotes systemic acquired resistance via azelaic acid and its precursor 9-oxo nonanoic acid — Supplementary Data 

# *Arabidopsis* ENHANCED DISEASE SUSCEPTIBILITY1 promotes systemic acquired resistance via azelaic acid and its precursor 9-oxo nonanoic acid

## Supplementary Data

Data files

**Files in this Data Supplement:**

- Supplementary Data - Supplementary Data
