# Supplementary material for: Arabidopsis ENHANCED DISEASE SUSCEPTIBILITY1 promotes systemic acquired resistance via azelaic acid and its precursor 9-oxo nonanoic acid
Source: J Exp Bot. 2014 Aug 11;65(20):5919–31. doi: 10.1093/jxb/eru331 (PMC4203127; doi:10.1093/jxb/eru331)
Supplement: Supplementary Data [file supp_eru331_jexbot127514_file001.pdf]

ID	theoretical mass [M-H]-	experimental mass [M-H]-	Error (ppm)	Annotated as	chemical formula
1	215.16527	215.1652916	-0.10	12-Hydroxydodecanoic acid	C12H24O3
2	221.081935	221.0819606	-0.12	Monoisobutyl phthalic acid	C12H14O4
3	243.066285	243.0663066	-0.09	3,3',4'5-Tetrahydroxystilbene	C14H12O4
4	253.050635	253.0506626	-0.11	Hispidol	C15H10O4
5	255.066285	255.0663186	-0.13	Isoliquiritigenin	C15H12O4
6	257.04555	257.0455956	-0.18	Gentisin	C14H10O5
7	269.04555	269.0455786	-0.11	Sulphuretin	C15H10O5
8	271.0612	271.0612396	-0.15	Naringenin chalcone	C15H12O5
9	273.07685	273.0768976	-0.18	Phloretin	C15H14O5
10	281.11832	281.1183736	-0.19	Randainol	C18H18O3
11	285.07685	285.0769156	-0.23	Oxypeucedanin	C16H14O5
12	287.056115	287.0561416	-0.09	Micromelin	C15H12O6
13	295.13397	295.1340366	-0.23	4-Prenylresveratrol	C19H20O3
14	301.071765	301.0718036	-0.13	Homoeriodictyol chalcone	C16H14O6
15	303.05103	303.0510896	-0.20	Pentahydroxyflavanone	C15H12O7
16	311.098375	311.0983406	0.11	Galactose-beta-1,4-xylose	C11H20O10
17	315.087415	315.0874016	0.04	Cajanol	C17H16O6
18	327.07216	327.0722216	-0.19	Bergenin	C14H16O9
19	403.285385	403.2854946	-0.27	MG(0:0/22:5(4Z,7Z,10Z,13Z,16Z)/0:0)	C25H40O4
20	405.11911	405.1192266	-0.29	Astringin	C20H22O9
21	415.10346	415.1035976	-0.33	Daidzin	C21H20O9
22	417.11911	417.1192536	-0.35	Barbaloin	C21H22O9
23	417.155495	417.1556186	-0.30	(+)-Syringaresinol	C22H26O8
24	421.114025	421.1141846	-0.38	Plicatic acid	C20H22O10
25	429.301035	429.3011466	-0.26	Convallamarogenin	C27H42O4
26	431.098375	431.0984716	-0.23	Vitexin	C21H20O10
27	431.13476	431.1348676	-0.25	2-(2,4,5-Trimethoxyphenyl)-5,6,7,8-tetramethoxy-4H-1-benzopyran-4-one	C22H24O9
28	433.114025	433.1141636	-0.32	Phlorizin chalcone	C21H22O10
29	435.09329	435.0933886	-0.23	Irisxanthone	C20H20O11
30	435.129675	435.1298096	-0.31	Phlorizin	C21H24O10
31	439.358155	439.3583166	-0.37	21beta-Hydroxyserrat-14-en-3-one	C30H48O2
32	445.114025	445.1141406	-0.26	Biochanin A-beta-D-glucoside	C22H22O10
33	445.332335	445.3324916	-0.35	3-Dehydroteasterone	C28H46O4
34	447.09329	447.0934396	-0.34	Carthamone	C21H20O11
35	447.129675	447.1296576	0.04	Neosakuranin	C22H24O10
36	449.10894	449.1090616	-0.27	2',3,4,4',6'-Peptahydroxychalcone 4'-O-glucoside	C21H22O11
37	457.36872	457.3688716	-0.33	Soyasapogenol B	C30H50O3
38	461.10894	461.1090806	-0.31	Isoscoparine	C22H22O11
39	463.12459	463.1247026	-0.24	Hesperetin 7-O-glucoside	C22H24O11
40	465.103855	465.1037886	0.14	Taxifolin 7-O-beta-D-glucopyranoside	C21H22O12
41	471.347985	471.3481476	-0.35	Gratiogenin	C30H48O4
42	473.363635	473.3637906	-0.33	Sapelin A	C30H50O4
43	477.103855	477.1039886	-0.28	Isorhamnetin 3-O-beta-D-glucopyranoside	C22H22O12
44	487.3429	487.3431176	-0.45	Asiatic acid	C30H48O5
45	489.35855	489.3587516	-0.41	Barringtogenol C	C30H50O5
46	491.119505	491.1196586	-0.31	Aurantio-obtusin beta-D-glucoside	C23H24O12
47	505.135155	505.1353836	-0.45	Junipegenin B 7-O-glucoside	C24H26O12
48	519.150805	519.1508646	-0.12	Chryso-obtusin glucoside	C25H28O12
49	519.18719	519.1873696	-0.35	Brusatol	C26H32O11
50	521.13007	521.1302706	-0.39	Iridin	C24H26O13
51	521.20284	521.2028166	0.04	Isobrucein A	C26H34O11
52	549.197755	549.1975366	0.40	Eucommin A	C27H34O12
53	561.37968	561.3799596	-0.50	Cholesterol glucuronide	C33H54O7
54	563.140635	563.1410386	-0.72	Apigenin 7-O-[beta-D-apiosyl-(1->2)-beta-D-glucoside]	C26H28O14
55	577.374595	577.3749046	-0.54	Asparagoside A	C33H54O8
56	609.146115	609.1464736	-0.59	Lucenin-2	C27H30O16

Supplemental Table S1 Annotated metabolites identified by FT-ICR-MS in SARiac1-3. The signal-to-noise (S/N) ratio of each mass in the HPLC fractions of extracts from *AvrRpm1-HA*-expressing wt plants was at least five-fold higher compared to their S/N ratio in the corresponding HPLC fractions of extracts from the *eds1-2* mutant. This experiment was repeated two times with similar results.

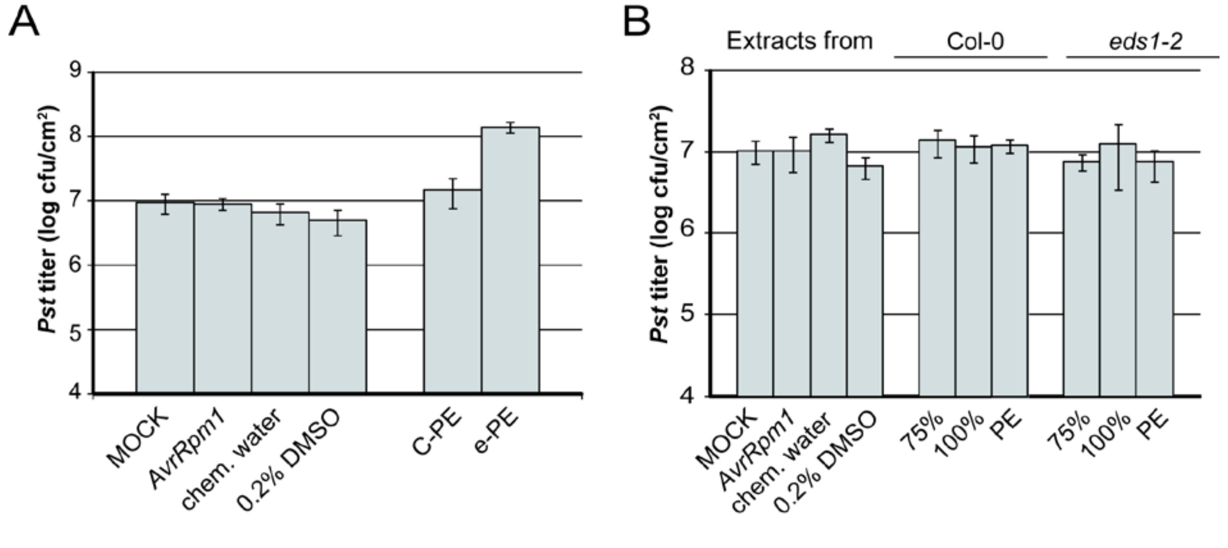

Supplemental Figure S1 SAR bio assays in *eds1-2* mutant plants (A/B) *eds1-2* mutant plants were locally treated with 10 mM $MgCl_2$ (MOCK), *Pst*/*AvrRpm1* (*AvrRpm1*), chemical-treated water (chem. water), 0.2% DMSO, or with different fractions from plants extracts. (A) Plant were treated with the PE-phases from DEX-treated *pDEX:AvrRpm1-HA* Col-0 (C-PE) and *pDEX:AvrRpm1-HA eds1-2* (e-PE) plants. (B) Plants were treated with the SPE eluates indicated below the panel (75% and 100% refer to MeOH) from DEX-treated *pDEX:AvrRpm1-HA* Col-0 and *pDEX:AvrRpm1-HA eds1-2* mutant plants as indicated above the panel. (A/B) Three days later, systemic leaves were infected with *Pst* and the resulting *Pst* titers are shown at four dpi. This experiment was repeated two times with similar results.

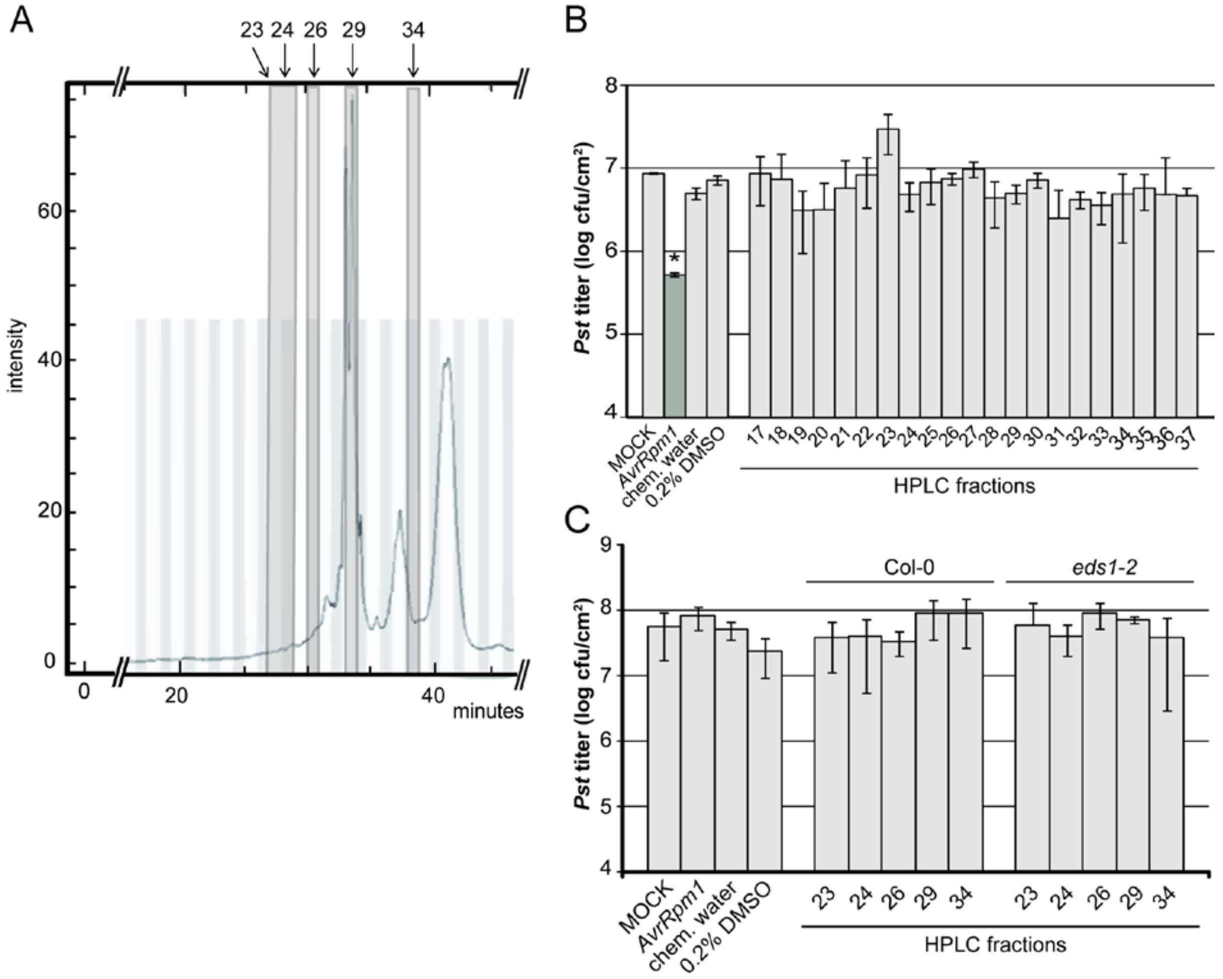

Supplemental Figure S2 HPLC-assisted separation of SAR-inducing metabolites and their dependency on *EDS1*. (A) UV absorption signal of a MeOH gradient HPLC chromatogram derived from DEX-treated *pDEX:AvrRpm1-HA eds1-2* plants. The signal intensity at 260 nm (y-axis) is shown against the HPLC retention time in minutes (x-axis). Fractions 17 to 37 are highlighted as alternating grey and white bars. The fractions that correspond to SAR-inducing fractions derived from wt plants are further highlighted in light grey and numbered above the panel. (B) SAR bio assay in Col-0 wt plants using the HPLC fractions from the *eds1-2* mutant for the primary treatment. Col-0 plants were locally treated with 10 mM $MgCl_2$ (MOCK), *Pst/AvrRpm1* (*AvrRpm1*), chemical-treated water (chem. water), or 0.2% DMSO as controls or with HPLC fractions 17 to 37 derived from DEX-treated *pDEX:AvrRpm1-HA eds1-2* mutant plants. (C) SAR bio assay in *eds1-2* mutant plants. *eds1-2* mutant plants were locally treated with the same controls as in (B) or with the SAR-inducing HPLC fractions (as indicated below the panel) from DEX-treated *pDEX:AvrRpm1-HA* Col-0 wt plants (as indicated above the panel) or the corresponding HPLC fractions from the *eds1-2* mutant. (B/C) Three days after the primary treatment, systemic leaves were infected with *Pst* and the resulting *Pst* titers are shown four days after infection (dpi). Asterisk above bar indicates a statistically significant difference to the MOCK control (* $P < 0.05$, Student´s *t* test). These experiments were repeated two (B/C) to three (A) times with similar results.

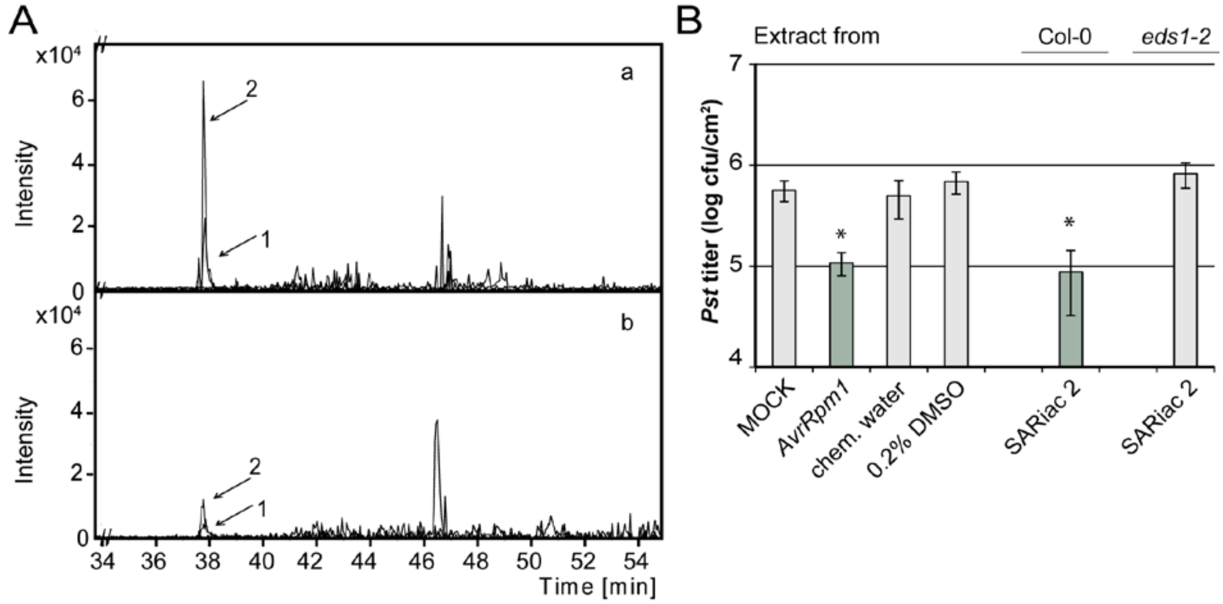

Supplemental Figure S3 The SAR-inducing activity of SARiac2 is associated with the accumulation of ONA and AzA. (A) LC-MS analysis of SARiac 2 from DEX-treated *pDEX:AvrRpm1-HA* Col-0 plants and the corresponding HPLC fraction from DEX-treated *pDEX:AvrRpm1-HA eds1-2* mutant plants. Intensity peaks (y-axis) detected in the negative ionization mode are plotted against the LC retention time in minutes (x-axis). Upper panel (a) corresponds to SARiac2 from wt plants and lower panel (b) to the corresponding HPLC fraction from the *eds1-2* mutant. *EDS1*-dependent accumulation was observed for mass 1 (9-oxo nonanoic acid; ONA) and 2 (azelaic acid; AzA). (B) SAR bio assay of SARiac 2. Col-0 plants were locally treated with 10 mM $MgCl_2$ (MOCK), *Pst*/*AvrRpm1* (*AvrRpm1*), chemical-treated water (chem. water), 0.2% DMSO, or with SARiac 2 from wt plants or the corresponding HPLC fraction from the *eds1-2* mutant. Three days later, systemic leaves were infected with *Pst* and the resulting *Pst* titers are shown at four dpi. Asterisks above bars indicate statistically significant differences to the MOCK or 0.2% DMSO controls (* $P < 0.05$, Student´s *t* test).

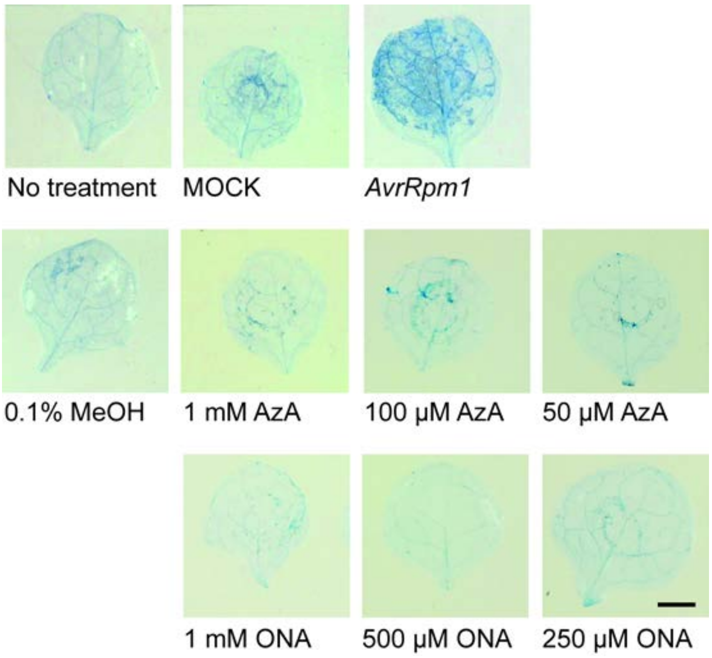

Supplemental Figure S4 Trypan blue staining of ONA- and AzA-treated leaves. Leaves of Col-0 plants were not treated or syringe-infiltrated as indicated below the micrographs with 10 mM $MgCl_2$ (MOCK), 0.1% Methanol (MeOH), or *Pst*/*AvrRpm1* (*AvrRpm1*) as controls or with 1 mM, 100 µM, or 50 µM of Azelaic acid (AzA) or 1 mM, 500 µM, or 250 µM of 9-oxo nonanoic acid (ONA). Three days post-infiltration, the leaves were analyzed by lacto-phenol Trypan blue staining. Representative micrographs are shown from 10 leaves of 5 different Col-0 plants that were analyzed per treatment. Bar = 2 mm.

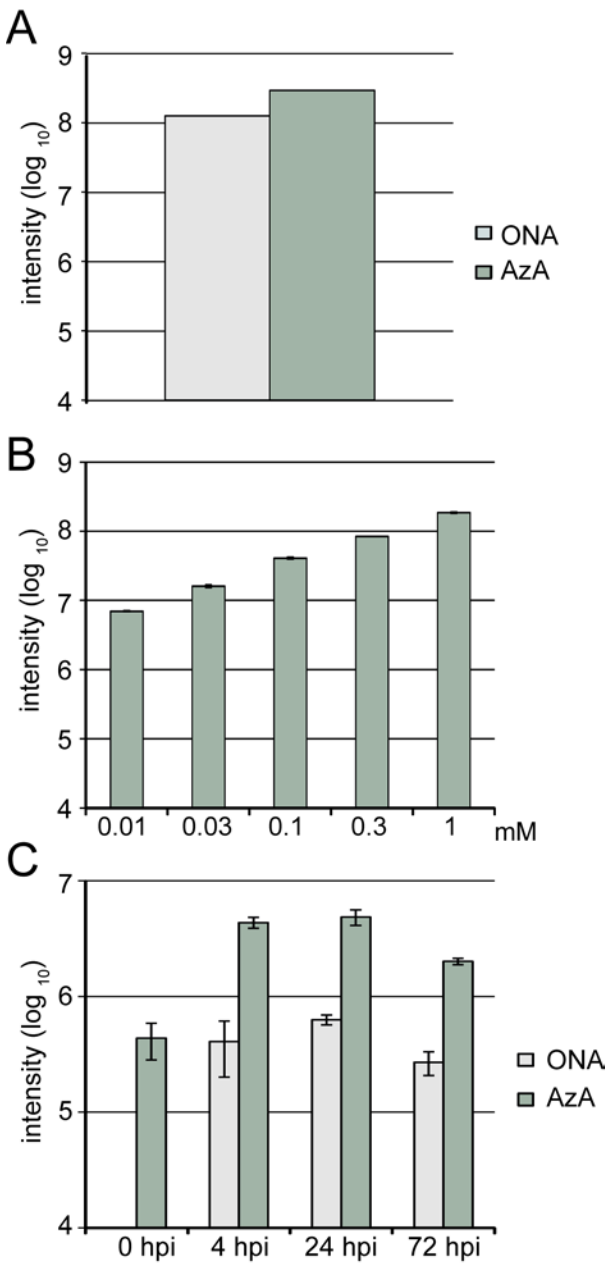

Supplemental Figure S5 LC-MS of ONA after storage at -80ºC and after infiltration into plants. (A) MS signal intensities of ONA (light grey bars) and AzA (dark grey bars) in an 8 mM stock solution that had been kept at -80ºC for ~three months. By extrapolation of an AzA standard curve (shown in B), the 8 mM stock contained ~1.4 mM of AzA. Thus, a signal intensity ratio of ~1:3 (ONA:AzA in A) corresponds to a molar ratio of ONA:AzA of ~6:1. (C) Integrity of exogenous ONA in *Arabidopsis* leaves. Col-0 leaves were untreated (0 hpi) or syringe-infiltrated with 250 µM ONA. The treated leaves were harvested at 4, 24, and 72 hpi. The samples were ground in liquid N_2, extracted with MeOH, and dried by evaporation to concentrate the metabolites that were dissolved in MeOH for LC-MS analysis. In contrast to AzA, ONA could not be detected in the samples from untreated Col-0 leaves. At 4 hpi the signal intensity ratio between ONA and AzA had shifted to ~1:10 (from 1:3 in the infiltrated solution diluted from the stock analyzed in A). This signal intensity ratio did not appear to change until 72 hpi. The data depicted in (C) includes measurements from two independent experiments that were repeated two times with comparable results using two different concentrations from the stock analyzed in (A).

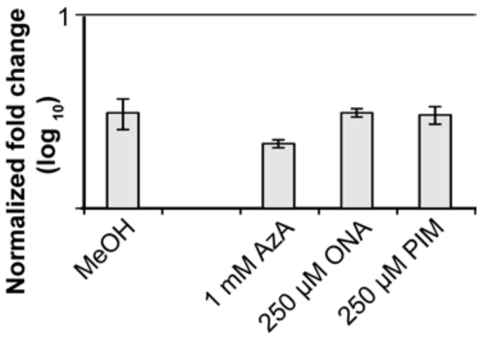

Supplemental Figure S6 Systemic *AZI1* expression in response to local ONA and AzA applications. Col-0 plants were locally treated with 0.1% methanol (MeOH), 1 mM AzA, 250 µM ONA, or 250 µM PIM. Three days later, RNA was isolated from systemic untreated leaves of the treated plants and analysed by qRT-PCR with primers specific for *AZI1* (Jung *et al.*, 2009). *AZI1* transcript accumulation was normalized to that of *TUBULIN.* The normalized fold change is shown relative to *AZI1* transcript accumulation in untreated plants. This experiment was repeated two times with similar results. The data shown here originate from the same samples that were used in Fig. 6A.
